# Functional Outcome and Hip Survival Rate in Traumatic Femoral Head Fractures

**DOI:** 10.3390/jcm15124741

**Published:** 2026-06-18

**Authors:** Christian Prangenberg, Thomas Loy, Alberto Alfieri Zellner, Jonas Roos, Sebastian Scheidt, Lisa Roder, Soufian Ben Amar, Kristian Welle

**Affiliations:** Clinic for Orthopedics and Trauma Surgery, University Hospital Bonn, 53127 Bonn, Germany; thomas.loy@ukbonn.de (T.L.); alberto.zellner@ukbonn.de (A.A.Z.); jonas.roos@ukbonn.de (J.R.); sebastian.scheidt@ukbonn.de (S.S.); lisa_fiona.roder@ukbonn.de (L.R.); soufian.ben_amar@ukbonn.de (S.B.A.); kristian.welle@ukbonn.de (K.W.)

**Keywords:** femoral head fracture, Pipkin, Harris Hip Score, polytrauma

## Abstract

**Objective:** Femoral head fractures are rare and severe injuries often associated with high-energy trauma. Early recognition and rapid, stable reduction are essential for successful treatment to prevent complications and morbidity. This retrospective study aims to describe and analyze long-term outcomes and survival rates of the femoral head after a Pipkin fracture. Methods: Between 2012 and 2021, all patients with a femoral head fracture who were treated in a Level I Trauma Center were assessed and analyzed. Two examiners performed a physical examination and radiological control of patients and called the patients for a final follow-up. Anterior and posterior fracture-dislocations and femoral head fractures were classified according to Pipkin’s classification system. The functional outcome was assessed using the Harris Hip Score (HHS). **Results:** Over a 10-year period, *n* = 15 patients were diagnosed with a femoral head fracture. All patients were male; the average age at admission was 41.3 years. The mean follow-up was 43.7 months ± 46 months. No complications occurred in three patients (20%). Twelve patients had complications. The most common complications were nerve lesions and posttraumatic osteoarthritis. Regarding the outcome, no data were available for two patients; one patient died, and two of four patients remained with a Girdlestone resection arthroplasty at the follow-up. The mean score in HHS was 76.69 ± 20.3 (mean ± standard deviation). **Conclusions:** An overall complication rate of 80% was observed; however, functional outcomes were generally moderate at final follow-up. These findings highlight the considerable risk of complications associated with femoral head fractures, particularly nerve injury and posttraumatic osteoarthritis. Notably, six of 15 patients no longer retained their native hip joint at the time of assessment. The study is a Level 2b study.

## 1. Introduction

Fractures of the femoral head are rare injuries that often occur in the setting of high-impact trauma [1]. The most common mechanisms of injury are road traffic accidents and the so-called dashboard injuries. When impacting the dashboard, the force is transmitted axially from the knee via the femur to the hip. Therefore, in up to 75% of cases, the injury is combined with an acetabular fracture and associated dorsal hip dislocation [2,3,4].

Pipkin conducted the first systematic case series and description of the injury in 1957 [5]. However, femoral head fractures were first described 100 years earlier in an autopsy study by Birkett in 1869 [6]. Pipkin divides the fracture into four types: types 1 and 2 differ in the insertion of the teres ligament. Type I is defined if the fracture involves a non-weight-bearing cartilage surface (below the round ligament insertion); type II is defined if it involves a weight-bearing area (above the round ligament insertion); type III is associated with a femoral neck fracture; type IV is associated with acetabular fractures [7]. In our cohort, 2/3 of the patients had a Pipkin type 4 fracture. There was no Pipkin 3 fracture in our cohort.

As CT diagnostics became increasingly established, a new classification, according to Chiron, was introduced in 2013 [8]. His system considers the size of the fragments and any associated acetabular wall or femoral neck fractures. The suggested course of action is based on the CT scan results after reducing the hip joint [8]. In everyday clinical practice, the fractures are classified according to the Pipkin classification. When treating Pipkin type 1 fractures, removing the fragments is a more effective procedure than open osteosynthesis; otherwise, cartilage damage can occur due to the exposed joint bodies [1]. However, open osteosynthesis has proven effective for Pipkin type 2–4 fractures [1].

In the literature, long-term follow-ups of 13 years for Pipkin 1 and 2 fractures show a good result in 89% of cases without any further complaints [3]. Large meta-studies show a better outcome for Pipkin 1 and 2 fractures after surgical therapy than after conservative therapy, so that, with a few exceptions, surgical treatment is always sought [1].

In 22% of cases, implantation of a hip prosthesis is necessary during treatment due to posttraumatic osteoarthritis [9]. In 13% of cases, this occurs within the first six months after trauma [9]. Therefore, in some studies, primary endoprosthetic treatment is recommended for patients over 65 years of age [3].

In addition to posttraumatic osteoarthritis, femoral head necrosis occurs in 12% of cases [2,10].

There is also a risk of heterotopic ossification. These occur in up to 46% of cases when an anterior approach is chosen [11]. Therefore, according to the literature, a dorsal approach should be preferred [3,11]. However, this can also lead to sciatic nerve lesions [11]. These are also described in the context of the dislocation event [11]. Other nerve lesions, such as the femoral nerve, are observed less frequently [11].

Recent literature, such as the study by Shakya et al. [10], confirms that femoral head fractures are rare injuries typically associated with high-energy trauma and a considerable risk of complications. They found a high rate of posttraumatic osteoarthritis in their cohort (33%). The functional outcome decreased with a complex fracture pattern following Pipkin classification. Furthermore, due to the lack of consensus regarding optimal management strategies, treatment approaches remain highly variable, highlighting the need for additional clinical data to guide decision-making.

However, to date, few long-term data have been collected at the time at which endoprosthetic joint replacement becomes necessary after trauma. This study aims to determine the time interval between trauma and the mean survival time of the affected joint. Furthermore, long-term results on complications, functional outcomes, and quality of life will be collected.

## 2. Material and Methods

A retrospective cohort study included all patients treated for proximal femur fractures at the Department of Orthopedics and Trauma Surgery, University Hospital Bonn, Germany, from 1 January 2012 to 1 January 2021. According to the Institutional Review Board of the University Hospital Bonn, Germany (No. 406/17), no additional ethical approval procedure is required for this retrospective analysis because no new data were collected and the study was based solely on the retrospective analysis of existing data from patient medical records.

For data collection, the institute’s clinical workplace system (KAS) and the patient file documented here were analyzed. The corresponding patients were identified using the ICD code for proximal femur fracture S72.08.

The inclusion criteria included treatment of a traumatic hip dislocation between 1 January 2012 and 1 January 2021.

Exclusion criteria included pertrochanteric fracture, underage patients, the presence of an underlying malignant disease, and metastases to the femur or pelvis.

A retrospective evaluation of the patient record collected data on the accident mechanism, concomitant injuries, time to operation, and complications during the inpatient stay. In addition, two independent specialists classified the fracture based on radiographs at admission according to Pipkin’s classification system. For dorsal acetabular fractures with posterior hip dislocation, a dorsal Kocher–Langenbeck approach was used. Particularly, in the case of preoperative neurology, a dorsal approach was used for open exposure and neurolysis of the sciatic nerve.

Furthermore, a retrospective evaluation of the routinely documented clinical follow-up examinations at 6 weeks and 12 months was performed. According to the standard institutional follow-up protocol, all patients underwent both clinical and radiological assessments at these time points. Clinical evaluation included a physical examination and assessment using the HHS score [12,13]. This score records pain, walking performance, and ability to cope with everyday life relative to a hip injury that has occurred. One hundred points can be achieved in the HHS. A score of 80–100 points indicates a good to excellent result, and a score below 70 is a poor result [12].

In addition, a native radiological X-ray control was performed. In case of a concomitant fracture of the acetabulum or the proximal femur, supplementary computed tomography (CT) imaging was performed.

For further follow-up, an additional telephone contact was made to evaluate the long-term outcome. The implantation of a hip prosthesis or the creation of a Girdlestone resection arthroplasty (GRA) was considered a failure of the hip joint.

Descriptive statistics were used to summarize demographic data and clinical scores. Mean values and standard deviations were calculated for continuous variables, while categorical variables were reported as frequencies and percentages.

**Figure 1:**
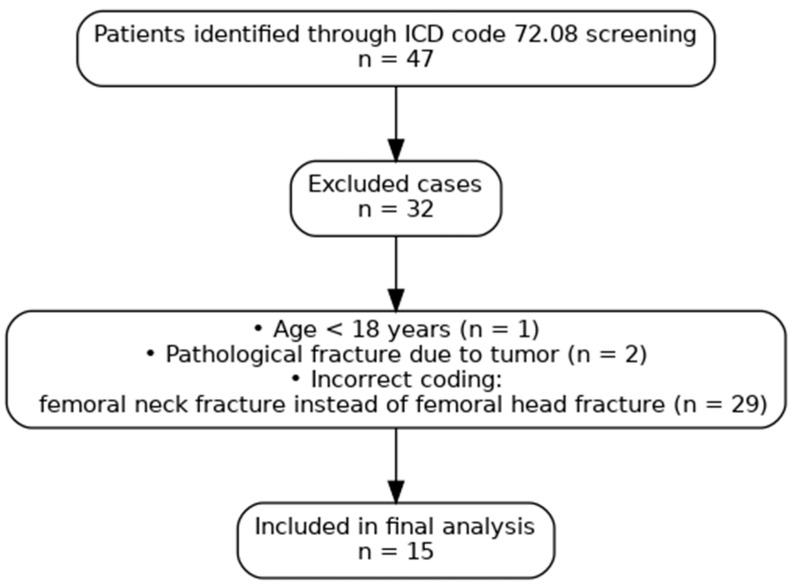
Flowchart of patient selection for the study population. Of 47 patients identified through ICD code 72.08 screening, 32 were excluded (1 patient aged < 18 years, 2 pathological fractures due to tumor, and 29 incorrectly coded cases), resulting in 15 patients included in the final analysis.

Comparisons between subgroups were performed using a one-way ANOVA. Correlation between muscle strength and clinical scores was analyzed using Pearson correlation coefficients. A *p*-value of <0.05 was considered statistically significant. All analyses were performed using SPSS Statistics version 26 (IBM Corp., Armonk, NY, USA) (Figure 1).

## 3. Results

Screening based on ICD code 72.08 identified a total of 47 cases during the study period. One patient was excluded because of an age below 18 years. Two additional patients were excluded because the femoral head fracture was pathological and associated with tumor-related bone disease. Of the remaining cases, 29 patients were excluded after chart and imaging review because the initial coding was incorrect and the diagnosis was femoral neck fracture rather than femoral head fracture. Ultimately, 15 patients fulfilled the inclusion criteria and were included in the final analysis.

The average age of the patients at admission was 41.3 years.

Two patients suffered a Pipkin type 1 fracture, three patients suffered a Pipkin type 2 fracture, and ten patients suffered a Pipkin type 4 fracture. There was no type 3 Pipkin fracture in the cohort.

Six patients suffered fractures in a car accident, and seven patients in a motorcycle accident. One patient had a fall at home; one patient was hit by a car.

Seven patients suffered polytrauma involving fractures of the spine and intra-abdominal injuries; three patients suffered an isolated extremity injury as a concomitant injury; two patients suffered an isolated serial rib fracture as a concomitant injury; three patients suffered no further injuries. Thirteen of 15 patients required intensive care treatment. The average intensive care stay was 6.87 days (±2.78 d). There were no significant differences between the groups *p* > 0.5.

On average, the patients were operated on 6.7 times due to the consequences of trauma. This includes 4.9 operations due to hip injuries. With an average of 9.4 (2.75) operations, patients with a Pipkin 4 fracture were operated on most frequently. An average of 7.1 hip operations were performed. Patients with Pipkin 1 and 2 fractures were operated on an average of two times (1.0). In Pipkin 1 fractures, all operations were performed on the hip. For Pipkin 2 fractures, 0.67 operations were performed on the hip. There were no significant differences between the groups *p* > 0.5.

Of the 15 patients operated on, a dorsal approach was chosen 11 times, a lateral approach three times and an anterior approach one time. Of the 15 patients, 14 patients received PAO prophylaxis with Ibuprofen, Etoroxcib or Indomethacin.

Six patients were treated with polypines, and three of those six patients also received a plate osteosynthesis of the acetabulum. Two patients received primary treatment of the acetabular fracture using plate osteosynthesis for a Pipkin 4 fracture. One patient received only a K-wire placement, which resulted in a peri-implant infection, resulting in GRA. One patient underwent a lag screw osteosynthesis, which subsequently had to be revised using THA implantation due to femoral head necrosis. One patient received primary treatment with THA, which had to be revised to a GRA due to an infection. One patient received a primary GRA in the presence of a posttraumatic hip joint infection. One patient underwent resection of a free joint body without refixation. One patient received labral fixation using Mitek anchors (DePuy Synthes, Zuchwil, Switzerland). One patient was treated conservatively.

No complications occurred in three patients. Three patients suffered a peroneal lesion postoperatively, and one patient suffered a sciatic nerve lesion. In three of four cases, this happened after a Pipkin 4 fracture. In one case, after a Pipkin 2 fracture. One patient suffered heterotopic ossification despite prophylaxis heterotopic ossification (PAO), as well as femoral head necrosis and a wound infection. Two patients suffered impaired wound healing. One patient suffered a breakout of the hip socket, resulting in a GRA, as mentioned above. Two patients suffered posttraumatic osteoarthritis (13.3%). Two patients suffered an intra-articular fracture fragment postoperatively; one was openly revised, and the second was left free of symptoms at the patient’s request.

**Figure 2:**
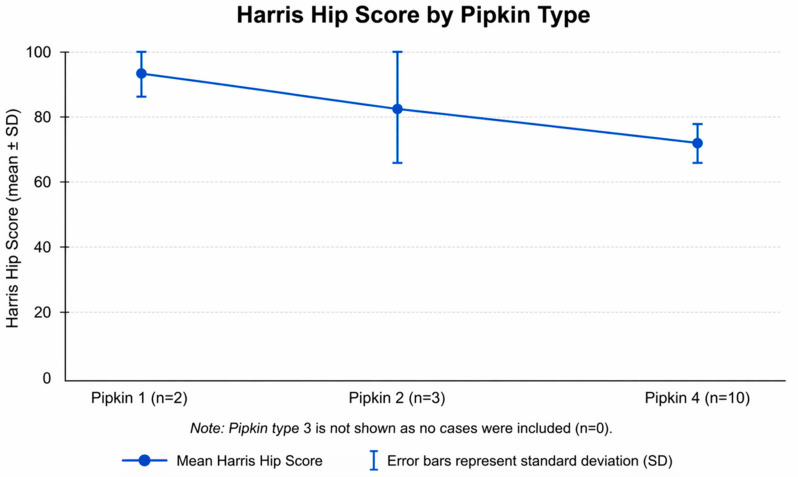
Harris Hip Score outcomes stratified by Pipkin classification, illustrating the relationship between functional hip results and fracture type severity; mean ± standard deviation.

Regarding the outcome, no data were available for two patients; one patient had died. Two patients still have impaired motor function due to a peroneal lesion or sciatic nerve lesion, six patients self-reported a condition with a good outcome, one patient is now free of symptoms after metal removal due to mechanical irritation, and another patient is still being treated for persistent wound seroma. Two of four patients still have a GRA. Two of four patients with a GRA received a total hip replacement. A total of four patients received hip prosthesis implantation, and two patients retained GRA. This means that six out of 15 patients had a failure of their native hip joint. On average, therapy failed after 26.67 months ± 42.66 (mean ± standard deviation). The mean score in HHS was 76.69 ± 20.3. Patients with Pipkin 1 fracture had a mean HHS score of 94 ± 6. Patients with a Pipkin 2 fracture had an average HHS score of 82.5 ± 17.5. Patients with a Pipkin 4 fracture had an average HHS score of 71.56 ± 6.8 (Figure 2). There were no differences in HHS between Pipkin 1, 2 or 4 fractures *p* = 1.

## 4. Discussion

Fractures of the femoral head are very rare fractures [3,14]. Often, only case series are described in the literature. Our study also shows a patient cohort of 15 patients. The first large meta-analysis of these case series was carried out by Bettinelli in 2021 [15]. Birkett first described these fractures in 1869 [6]. However, the first systematic classification was not carried out until 1957 by Pipkin [5].

In the majority of cases, these injuries occur in young patients due to high-speed trauma [14,16,17,18,19]. Patients often suffer polytrauma [15,18,19]. In our cohort, 14 of 15 had high-speed trauma. In our cohort, 86% of patients were injured due to a traffic accident. This finding is consistent with the description of Lehmann [3]. This mainly results in an impaction of the femoral head into the acetabulum, which is why most patients suffer from a Pipkin type 4 fracture [3]. This often leads to a dorsal hip joint dislocation [3,4,18,19,20,21].

This impaction poses a great risk for subsequent subchondral oedema of the femoral head and could be responsible for the relatively high number of six out of 15 patients who had a failure of their native hip joint in the first two years in our cohort.

If the mechanism of injury is low-energy trauma, an underlying pre-existing condition should be considered; in our cohort, the only patient with a low-energy trauma had osteoporosis, which was considered the contributing cause of the fracture.

The optimal treatment is controversial, and the choices include non-surgical, surgical excision of fragments, surgical internal fixation of fragments or primary arthroplasty [1,15].

In his cohort, Lehmann et al. generally require endoprosthetic treatment for patients over 65 [3,16].

In our cohort, the choice of therapy was made according to the available Pipkin classification, regardless of age. This meant that all described therapeutic procedures could be observed in our cohort. However, only one patient was treated conservatively. He had a Pipkin 1 fracture and a good outcome. This is consistent with the good results described in the literature for Pipkin 1 and 2 fractures [1,22]. Most of our patients (*n* = 14, 93.3%) received surgical treatment; Chen et al. have explored the surgical and conservative treatment options in a randomized controlled trial and have concluded that closed reduction and surgical treatment is better than closed reduction alone for Pipkin type I fractures [23]. The meta-analysis of the literature conducted by Bettinelli et al., which included 15 studies and a total of 274 cases, underlines the importance of surgical intervention and further states that fragment excision alone has a less favorable outcome compared to open reduction and internal fixation (ORIF) of the fragments.

Moreover, conservatively treated patients showed a lower satisfaction rate [17]. In our cases, the only time that we opted for a conservative treatment plan was because the patient was in critical condition due to polytrauma. Other than the Pipkin fracture, the patient suffered from a pulmonary laceration with pneumothorax on the left side, splenic rupture, renal laceration on the left side, hepatic laceration, and fracture of the fifth thoracic vertebra (AO A3). In the rest of the cases, we performed ORIF to achieve the best result possible. Interestingly, a review conducted by Tsai and colleagues, which included a total of eight studies and 97 patients, states that different therapies can be used to aim for specific outcomes. For instance, it seems like fragment excision alone yields better function, conservative treatment poses the highest risk for posttraumatic arthritis, and ORIF has the highest avascular necrosis risk [23].

The worst outcome in the literature is described for the Pipkin type III fracture. A complete failure rate is described here with high rates of femoral head necrosis and subsequent necessary endoprosthetic treatment [1,22]. No Pipkin type 3 fractures could be observed in our cohort. The greatest challenge, however, is the treatment of Pipkin 4 fractures. The literature describes an almost complete need for surgical treatment, as the present acetabular fracture usually had to be treated using plate osteosynthesis [15].

In our study, all Pipkin 4 fractures were treated surgically. This is consistent with the results described in the literature [15]. Pipkin 4 fractures underwent surgery most frequently compared to the Pipkin 1 and Pipkin 2 fractures, with an average of 7.1 hip operations. Overall, patients with Pipkin 4 fractures were treated surgically 9.4 times. Patients with a Pipkin 4 fracture were significantly more likely to be polytraumatized, which is why several operations were necessary due to further injuries.

A further large percentage of patients who encountered complications suffered from posttraumatic arthritis of the hip joint or avascular necrosis of the femoral head. These complications ultimately led to the endoprosthetic treatment that we try to avoid in young patients due to the known longevity rates and complications that these procedures can include [24]. In most cases, these injuries occur in young patients due to high-speed trauma, which often results in an impaction of the femoral head into the acetabulum, which is why most of the patients suffer a Pipkin type 4 fracture. We speculate that this impaction poses a great risk for subsequent subchondral oedema of the femoral head and could be responsible for the relatively high number of patients undergoing prosthetic hip surgery in the following months.

The incidence of femoral head necrosis and heterotopic ossification in our cohort was consistent with rates reported in the existing literature.

A possible reason for the high number of complications could be that *n* = 6 (37.5%) suffered a femoral head lesion while being polytraumatized.

Measured in our patient population, the complications of femoral head necrosis and heterotopic ossification occurred by the quantity stated in the available literature [15].

Our patient was now shown to have heterotopic ossification. This corresponds to significantly lower rates than the 20% described in the literature [15,22]. This may be because our patients received prophylaxis with Ibuprofen, Etoricoxib or Indomethacin [25].

In addition to the complications already mentioned in the literature, we identified wound infections, peri-implant infections, and nerve lesions as essential complications. In this case, four of 15 patients suffered postoperative nerve damage. In three of four cases this occurred after a Pipkin 4 fracture. In one case, a Pipkin 2 fracture. A possible cause for this can be the accompanying hip dislocation and the resulting stretching damage. This is consistent with the better results described in the literature with prompt reduction in a hip dislocation [18,19].

Another quarter of the patients was finally discharged with a GRA following an infection as a complication. Secondary surgical intervention was needed in 14.5% of the patients in a study by Sen et al., which lies well below our reported figure of postoperative infections [26]. In a narrative review by Menger et al., postoperative infection is named one of the most common complications, with an occurrence of 3.2% [19].

In our study, two patients presented with impaired wound healing. Both patients suffered a Pipkin 4 fracture. One reason may be the significantly complex injury pattern and the substantially higher number of operations. A possible reason for the high number of complications could be that six of 15 patients suffered a femoral head lesion because of polytrauma.

Overall, postoperative nerve damage was seen in about one quarter of the cases and a temporary GRA in about another quarter. This means that these two complications appeared in half of the cases. In addition, we could observe that within 24 months, six of 15 patients no longer had their own hip joint (GRA and arthroplasty).

In meta-analyses, the size of the fracture fragments and their exact reduction are seen as the most crucial prerequisite for a good outcome [15]. In our view, as the results of hip endoprosthetics become increasingly better, immediate endoprosthetic treatment instead of osteosynthesis must be considered, especially in complex fractures in which exact reduction is not possible, and this is mainly the case in the presence of a Pipkin 4 fracture. However, there is still a lack of data in the literature, so this hypothesis still needs to be examined in further studies.

### Functional Outcome

In this study, we collected functional outcome data. The mean Harris Hip Score (HHS) was 76.69 ± 20.3. Clinical outcomes indicated that HHS decreased with increasing fracture severity according to the Pipkin classification. Treatment strategies for these fractures are highly heterogeneous and largely depend on associated injuries. Moreover, our patient cohort was small and inherently heterogeneous, which limits comparability with other studies, as each case presents unique characteristics.

In young polytraumatized patients with concomitant thoracic or abdominal injuries, femoral head fractures are often not the immediate clinical priority. This may delay surgical intervention and negatively affect functional outcomes. Additionally, 13 patients in our cohort required intensive care unit (ICU) treatment, which is associated with an increased risk of periarticular ossifications [27,28]. This introduces the possibility of a confounding effect from heterotopic ossification (HO), as HO can substantially influence clinical outcomes and the assessment of treatment efficacy, independent of the initial trauma and potentially related instead to ICU management.

In line with the findings reported by Shakya et al. [10], our results confirm that femoral head fractures are associated with substantial complication rates, including avascular necrosis, posttraumatic osteoarthritis, and heterotopic ossification. However, differences were observed in functional outcomes. While Shakya et al. [10] reported overall favorable Harris Hip Scores, the mean HHS in Pipkin I and II fractures in our cohort was higher compared to the cohort of Shakya et al. [10]; in patients with higher-grade Pipkin fractures, the HHS was similar. This may come from the higher amount of Pipkin IV fractures (*n* = 10) in our cohort and smaller sample size of low-grade fractures. The decrease in HHS with increasing fracture complexity according to the Pipkin classification was observed both in our cohort and in Shakya et al. [10]. Consequently, direct comparison between studies remains limited.

Limitations of this study are small sample size, a heterogeneous patient cohort and confounding due to the multitude of treatment options.

**Figure 3:**
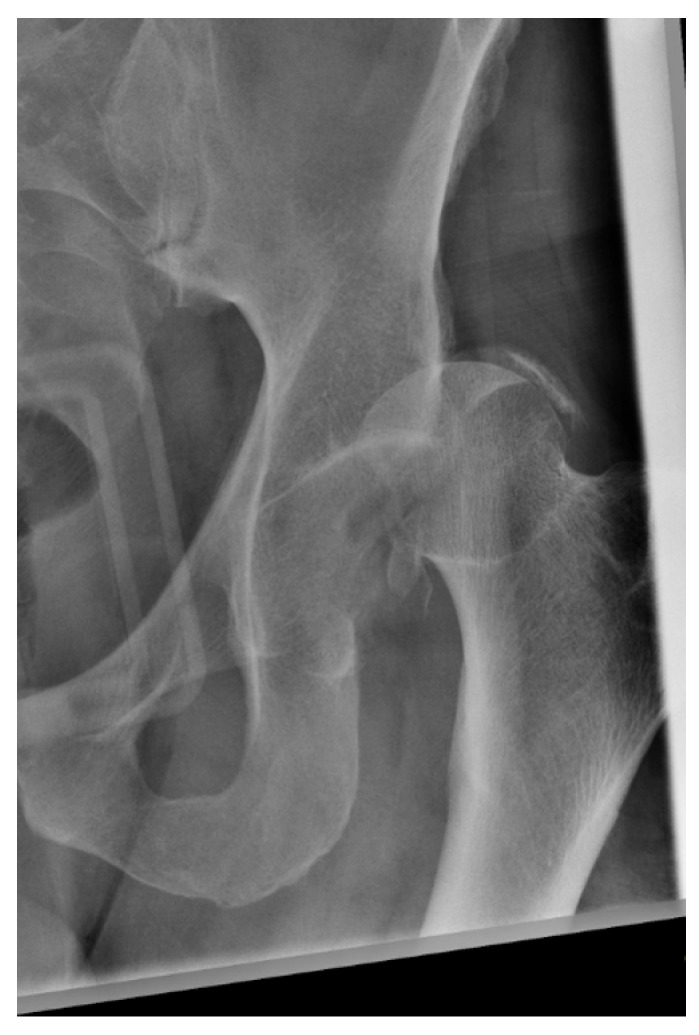
AP X-ray of a pelvis showing a Pipkin 4 fracture involving the acetabulum with a hip dislocation in the shock room.

**Figure 4:**
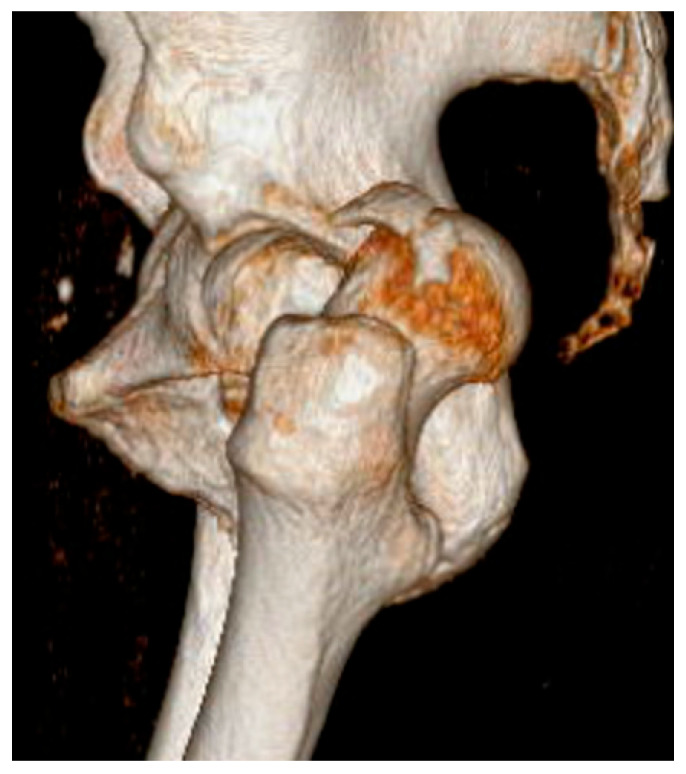
3D reconstruction of the CT of the Pipkin 4 fracture with hip dislocation.

**Figure 5:**
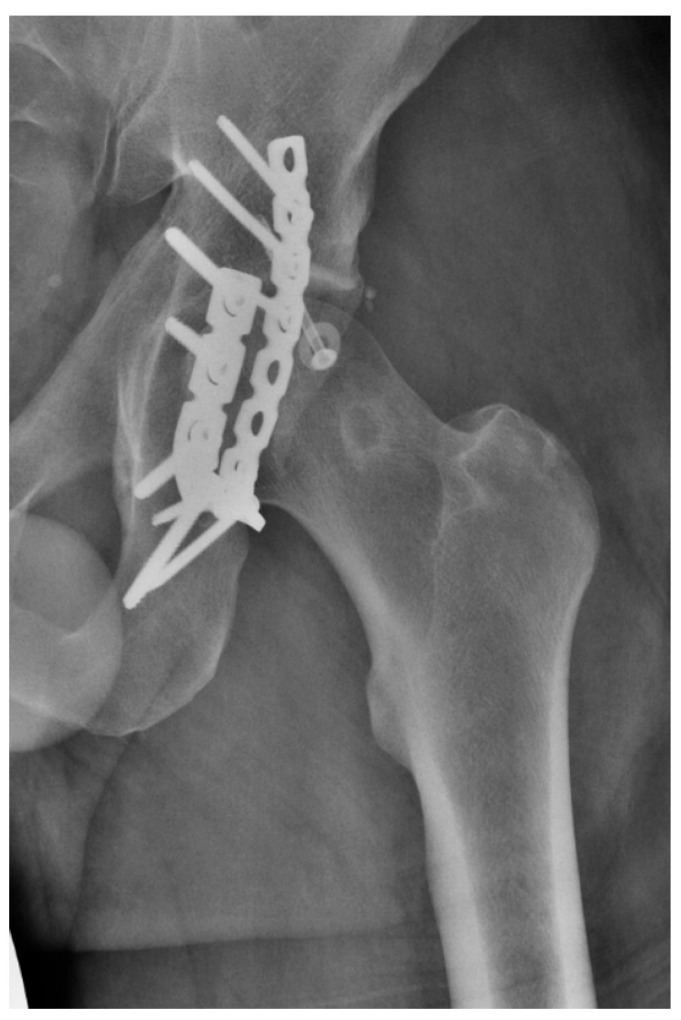
AP X-ray of a pelvic overview 6 weeks postoperatively after open retrieval of the free joint body and stabilization of the acetabular fracture using a two-plate osteosynthesis.

**Figure 6:**
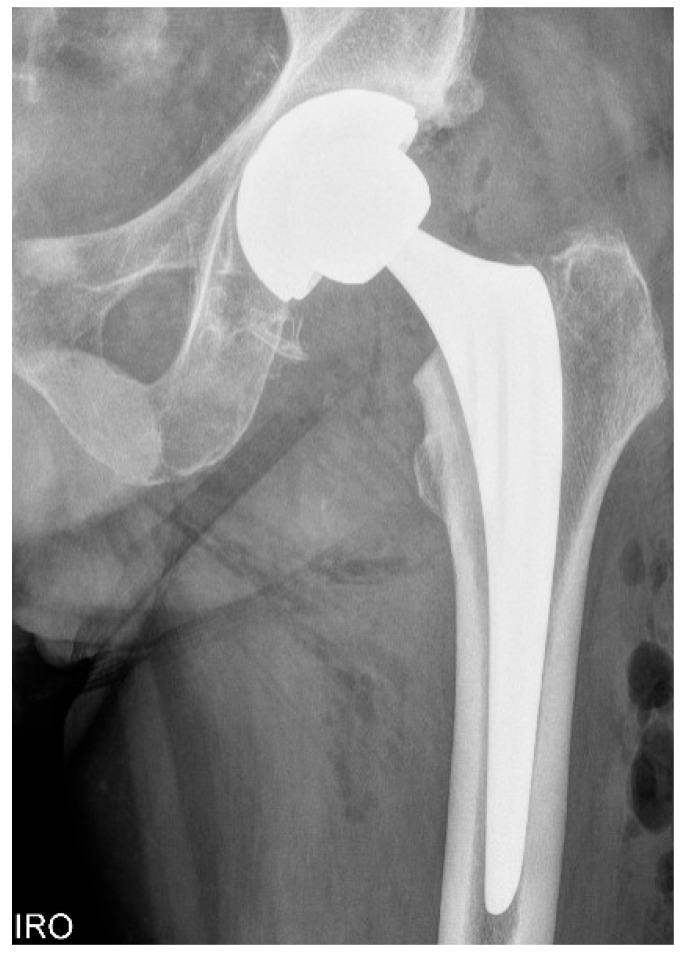
AP X-ray of a pelvic overview 12 weeks after implantation of a cementless hip prosthesis after femoral head necrosis.

**Figure 7:**
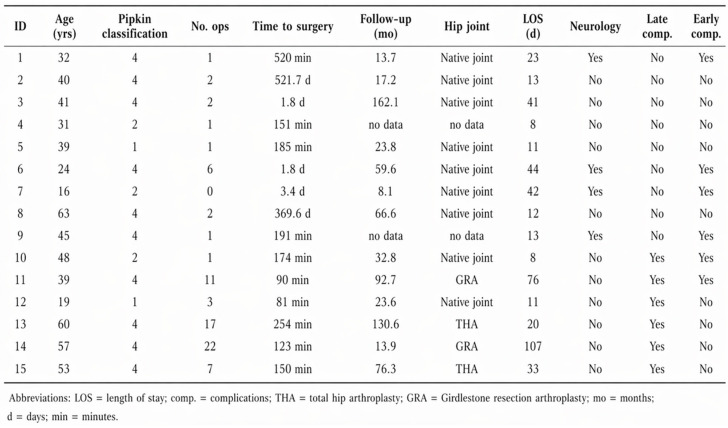
Individual patient characteristics showing fracture type according to Pipkin classification and total number of hip-related surgical procedures performed.

To enhance the reliability and applicability of future research, more extensive studies with more homogeneous patient groups and standardized treatment protocols are needed. A workaround for this problem could be conducting a multicenter study within the established trauma network of the Rhine Valley to increase the sample size and diversity of the patient population, thereby improving the generalizability of the results and providing more robust evidence for the optimal management of Pipkin fractures. In conclusion, the limitations of this study (small sample size, heterogeneous patient cohort, confounding due to the multitude of treatment options) necessitate a cautious interpretation of the study’s findings (Figure 3, Figure 4, Figure 5, Figure 6 and Figure 7).

## 5. Conclusions

Reviewing our study’s data, we found that most fractures of this unusual type were caused by high-energy trauma. However, when such fractures occur following a low-energy mechanism, an underlying pre-existing condition should be considered. In addition to the complications already mentioned in the literature, we were able to identify wound infections, peri-implant infections, and nerve lesions as important complications.

Even though femoral head fractures are a scarce type of fracture, the high number of complications shows that further studies are necessary to improve the outcome of the standard treatments.

## Data Availability

The raw data supporting the conclusions of this article will be made available by the authors on request.
